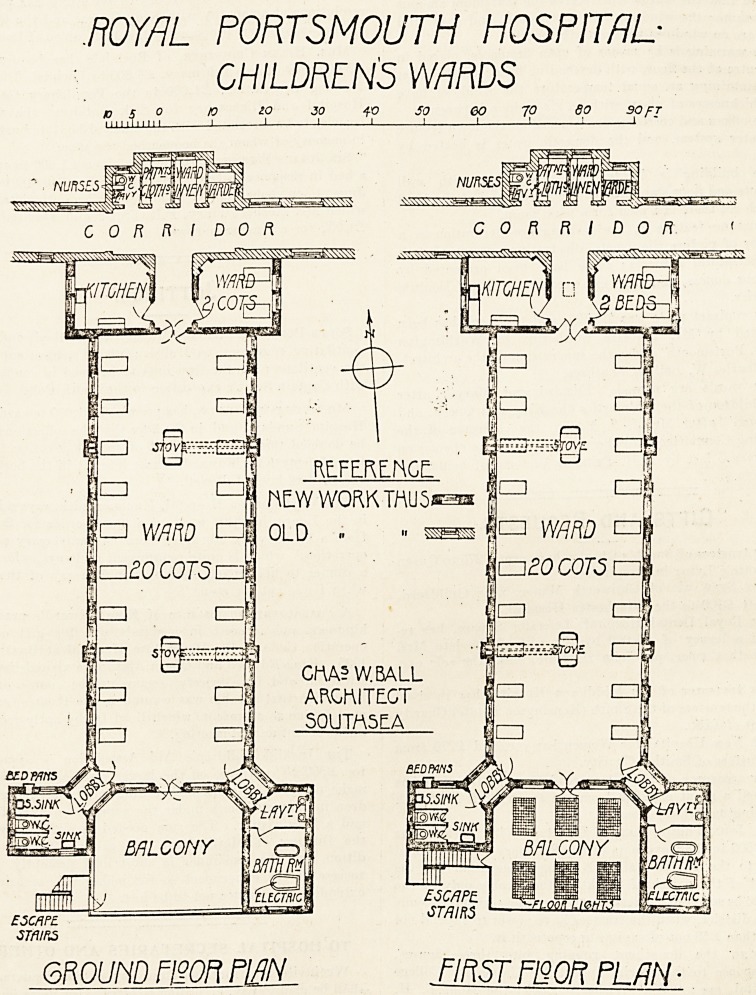# New Children's Wards at the Royal Portsmouth, Portsea, and Gosport Hospital

**Published:** 1910-06-04

**Authors:** 


### NEW CHILDREN'S WARDS AT THE ROYAL PORTSMOUTH, PORTSEA, AND GOSPORT HOSPITAL.

The new block containing the two children's wards is a continuation of the scheme commenced some 12 years ago for the rebuilding of the hospital. The block is two storeys high, and will accommodate 44 patients—20 in each of the two main wards and two in each of the two small wards—and there is room for four additional cots for babies in each of the main wards.

There is a ward kitchen on each floor at the northern end, and two lavatory spurs at the southern end, containing an electrical bath and the usual sanitary appliances, and between the spurs are covered balconies on to which the cots are wheeled when the weather is suitable.

The floors throughout are of fire-resisting construction, and are laid with Venetian terrazzo paving. The walls of the sanitary spurs and balconies are built of ivory-white glazed bricks, with green bands to form dado and skirting, and the walls of the wards and kitchens are finished in Keene's cement, and enamelled with pearline in two artistic greens, and the woodwork throughout is enamelled white. The wards are well lighted with a window on either side of each cot, and the building is so planned that the wards will receive the maximum amount of sunshine; the windows face east, south, and west, and there are no windows with a north aspect.

The warming is by means of open fireplaces, placed in the centre of the floor, with descending flues; and to assist in maintaining an equal temperature the windows have two thicknesses of glass, with an air-cushion between.

The offices and corridors are warmed on the low-pressure hot-water system, and the domestic water is heated by steam.

The building is lighted by electricity, with wall brackets and floor standards in the wards, and at the head of each cot there is a plug for a hand lamp.

A unique feature in the wall-surface decoration is a number of picture tile-panels, illustrating nursery rhymes and Scriptural subjects; these have been presented by generous donors, and were executed at the Royal Doulton Potteries.

The contract price was £5,320, and the work has been executed by Mr. Maurice Coltherup, of Warblington Street, Portsmouth, under the supervision of the architect, Mr. Charles W. Ball, of Southsea.

The wards are named "Edward and Mary," after two children of their Majesties the King and Queen, and "Young," after Mr. J. J. Young, the chairman of the Building Committee, and the new building was opened on February 3 last by H.H. Princess Victoria of Schleswig-Holstein.

## Figures and Tables

**Figure f1:**